# Supplementary material for: QKAN: quantum Kolmogorov-Arnold networks with applications in machine learning and multivariate state preparation
Source: npj Quantum Inf. 2026 Mar 11;12(1):73. doi: 10.1038/s41534-026-01202-5 (PMC13111150; doi:10.1038/s41534-026-01202-5)
Supplement: Supplementary file 1 — Supplementary Information [file 41534_2026_1202_MOESM1_ESM.pdf]

Supplementary Information for "QKAN: Quantum Kolmogorov-Arnold Networks with Applications in Machine Learning and Multivariate State Preparation"

Petr Ivashkov,[1,2,*] Po-Wei Huang,[1,3] Kelvin Koor,[1] Lirandë Pira,[1,†] and Patrick Rebentrost[1,4,‡]

[1] *Centre for Quantum Technologies, National University of Singapore, Singapore*
[2] *Department of Information Technology and Electrical Engineering, ETH Zürich, Zürich, Switzerland*
[3] *Mathematical Institute, University of Oxford, Oxford, United Kingdom*
[4] *Department of Computer Science, National University of Singapore, Singapore*

CONTENTS

I. Supplementary Note 1. Cloning diagonal block-encodings 1

II. Supplementary Note 2. Resource analysis and error propagation 2

III. Supplementary Note 3. Output estimation of CHEB-QKAN 4

IV. Supplementary Note 4. Multivariate Gaussian state preparation 4
A. Multivariate grid encoding 5
B. Polynomial approximation to exponential decay $e^{-\beta(x+1)}$ 6
C. Bounding the filling fraction of a D-dimensional Gaussian 6
D. Gate and qubit complexity of Gaussian state preparation 8

V. Supplementary Note 5. Generalized state preparation via CHEB-QKAN 9

References 10

I. SUPPLEMENTARY NOTE 1. CLONING DIAGONAL BLOCK-ENCODINGS

We show the simple fact that diagonal block-encodings can be duplicated across additional registers by tensoring with the identity:

Lemma I.1 (Cloning diagonal block-encodings). *Let U_x be an (α, a, ε)-block-encoding of* $\mathrm{diag}(x_1, \ldots, x_N)$. *Then $U_x \otimes I_k$ is an (α, a, ε)-block-encoding of*

$$\mathrm{diag}(x_1, \ldots, x_N) \otimes I_k = \mathrm{diag}(\underbrace{x_1, \ldots, x_1}_{K}, \ldots, \underbrace{x_N, \ldots, x_N}_{K}). \tag{1}$$

Proof. By definition, $\| \mathrm{diag}(x_1, \ldots, x_N) - \alpha(\langle 0|_a \otimes I_n) U_x (|0\rangle_a \otimes I_n)\| \leq \varepsilon$. Therefore,

$$\begin{aligned}
&\| \mathrm{diag}(x_1, \ldots, x_N) \otimes I_k - \alpha(\langle 0|_a \otimes I_n) U_x \otimes I_k (|0\rangle_a \otimes I_n)\| \\
=&\| \mathrm{diag}(x_1, \ldots, x_N) \otimes I_k - \alpha(\langle 0|_a \otimes I_n) U_x (|0\rangle_a \otimes I_n) \otimes I_k\| \\
=&\| \mathrm{diag}(x_1, \ldots, x_N) - \alpha(\langle 0|_a \otimes I_n) U_x (|0\rangle_a \otimes I_n)\| \leq \varepsilon.
\end{aligned}$$

Hence, $U_x \otimes I_k$ is an (α, a, ε)-block-encoding of $\mathrm{diag}(x_1, \ldots, x_N) \otimes I_k$, as claimed. □

* pivashkov@ethz.ch
† lpira@nus.edu.sg
‡ patrick@comp.nus.edu.sg

Step	*Gates*	*Auxiliary qubits*
DILATE	$C_x^{(l)}$	$a_x^{(l)}$
CHEB	$dC_x^{(l)}$	$a_x^{(l)} + 1$
MUL	$dC_x^{(l)} + C_w^{(l)}$	$a_x^{(l)} + 1 + a_w^{(l)}$
LCU	$\frac{d^2}{2} C_x^{(l)} + dC_w^{(l)}$	$a_x^{(l)} + 1 + a_w^{(l)} + \log_2(d+1)$
SUM	$\frac{d^2}{2} C_x^{(l)} + dC_w^{(l)}$	$a_x^{(l)} + 1 + a_w^{(l)} + \log_2(d+1) + \log_2 N^{(l)}$
	$\underbrace{\qquad}_{=C_x^{(l+1)}}$	$\underbrace{\qquad}_{=a_x^{(l+1)}}$

TABLE I. *Resource estimate for the $(l+1)$-th CHEB-QKAN layer.* We estimate the gate complexity $C_x^{(l+1)}$ and qubit requirements $a_x^{(l+1)}$ for constructing the $(l+1)$-th layer based on the corresponding quantities of the l-th layer and the cost $C_w^{(l)}$ of block-encoding the weight vectors. This recursive approach is used because each layer's block-encoding serves as the primitive building block for constructing the subsequent layer. The resource requirements are presented cumulatively, with costs increasing for each step. The final *SUM* step indicates the total accumulated cost, in terms of gates and auxiliary qubits, for realizing $l+1$ layers. Auxiliary qubits are required at each step to encode the current transformation.

II. SUPPLEMENTARY NOTE 2. RESOURCE ANALYSIS AND ERROR PROPAGATION

In this supplementary section, we provide a detailed analysis of the number of gates and auxiliary qubits for a multilayer CHEB-QKAN. Consider a CHEB-QKAN with L layers, where $N^{(l)}$ denotes the number of nodes in the l-th layer, and $N^{(0)}$ represents the input dimension. We assume that all activation functions in each layer are linear combinations of Chebyshev polynomials of degree at most d. Let $C_x^{(l)}$ represent the gate complexity of constructing a controlled diagonal block-encoding corresponding to the output of the l-th layer, with $C_x^{(0)}$ being the gate complexity of constructing a controlled diagonal block-encoding of the input. Additionally, let $C_w^{(l)}$ denote the gate complexity of constructing a controlled diagonal block-encoding of weights for the l-th layer. In Table I we estimate the gate complexity of constructing a diagonal block-encoding of the output of the $l+1$-th layer in terms of the number of applications of $C_x^{(l)}$ and $C_w^{(l)}$, which depend on the block-encoding methods used for the input and weights.

Constructing a controlled version of the obtained block-encoding, which is required for the next layer, can be achieved by controlling every gate in the block-encoding construction. This process has the same asymptotic complexity as the uncontrolled block-encoding, with an additional overhead of at most $\mathcal{O}(n_{\text{total}}^2)$ one- and two-qubit gates, where n_{total} is the total number of qubits [1]. Furthermore, here, we do not explicitly consider the additional one- and two-qubit gate overhead, which is upper bounded by $\mathcal{O}(n_{\text{total}}^2)$, and arises from decomposing multi-controlled operations and other steps in the construction. The reason for this omission is that we expect the dominant gate cost to come from constructing the diagonal block-encodings for inputs and weights, rather than from these additional overheads.

Table I presents the cumulative gate complexity and qubit requirements for each step in the construction of the $(l+1)$-th KAN layer. The auxiliary qubits in Table I are necessary for the block-encoding of the current transformation. Since the transformations are performed sequentially, each subsequent transformation adds to the gate and qubit cost. Consequently, the final transformation, denoted as the *SUM* step in Table I, represents the total cost of realizing the $l+1$ layers.

To estimate the gate and qubit complexity of an L-layer CHEB-QKAN in terms of $C_x^{(0)}$ and $C_w^{(l)}$, we recursively expand the gate cost and obtain the following expression for $C_x^{(L)}$:

$$C_x^{(L)} = \frac{d^2}{2} C_x^{(L-1)} + dC_w^{(L-1)}$$

$$
\begin{aligned}
&= \frac{d^4}{4} C_x^{(L-2)} + \frac{d^3}{2} C_w^{(L-2)} + d C_w^{(L-1)} \\
&= \frac{d^6}{8} C_x^{(L-3)} + \frac{d^5}{4} C_w^{(L-3)} + \frac{d^3}{2} C_w^{(L-2)} + d C_w^{(L-1)} \\
&\vdots \\
&= \left(\frac{d^2}{2}\right)^L C_x^{(0)} + d \sum_{l=1}^{L} \left(\frac{d^2}{2}\right)^{l-1} C_w^{(L-l)}.
\end{aligned}
$$

The total number of auxiliary qubits, a_x^L, can be expressed as:

$$
a_x^{(L)} = a_x^{(L-1)} + 1 + a_w^{(L-1)} + \log_2(d+1) + \log_2 N^{(L-1)} = a_x^{(0)} + \sum_{l=1}^{L-1} \left(1 + a_w^{(l)} + \log_2(d+1) + \log_2 N^{(l-1)}\right). \quad (2)
$$

In summary, a L-layer CHEB-QKAN requires $\mathcal{O}\left(d^{2L}/2^L\right)$ applications of controlled block-encodings of the input and weights and a number of auxiliary qubits that grow linearly with L.

To assess the error propagation in a multi-layer QKAN, recall that by Theorem 1 in the main text we can create a block-encoding of the single-layer vector $\Phi(\boldsymbol{x})$ up to the error $4d\sqrt{\varepsilon_x} + \varepsilon_w$. Suppose we could encode weights perfectly, i.e. $\varepsilon_w = 0$. Then, starting with the $(1, a_x, \varepsilon_x)$ block-encoding U_x of the input vector $\boldsymbol{x}$, the error in the first few layers evolves as

$$
\varepsilon_x \longrightarrow 4d\sqrt{\varepsilon_x} \longrightarrow 4d\sqrt{4d\sqrt{\varepsilon_x}} \longrightarrow \dots \quad (3)
$$

Consequently, after $L \geq 1$ layers, the accumulated error ε_{tot} in the block-encoding is

$$
\varepsilon_{\text{tot}} = \varepsilon_x{}^{(1/2)^L} (4d)^{1+\frac{1}{2}+\frac{1}{4}+\dots+2^{-L}} = \mathcal{O}\big(d^2\, \varepsilon_x{}^{(1/2)^L}\big). \quad (4)
$$

As a result, such recursive error propagation quickly amplifies any nonzero error. To achieve a constant small error in the output, $\varepsilon_{\text{tot}} = O(1)$, the input block-encoding must satisfy $\varepsilon_x = O\big(d^{-2^L}\big)$, i.e., decaying super-exponentially in L. The same analysis applies if we consider noiseless input ($\varepsilon_x = 0$) and track weight error ε_w. In the first few layers, the output error evolves as

$$
\varepsilon_w \longrightarrow 4d\sqrt{\varepsilon_w} + \varepsilon_w \longrightarrow 4d\sqrt{4d\sqrt{\varepsilon_w} + \varepsilon_w} + \varepsilon_w \longrightarrow \dots \quad (5)
$$

and after $L \geq 1$ layers the accumulated error ε_{tot} can be upper-bounded by

$$
\varepsilon_{\text{tot}} \leq 16d^2 \sum_{k=0}^{L-1} \varepsilon_w{}^{(1/2)^k} = \mathcal{O}\big(d^2\, \varepsilon_w{}^{(1/2)^L}\big). \quad (6)
$$

Similarly, to achieve $\varepsilon_{\text{tot}} = O(1)$ the weight block-encoding error must satisfy $\varepsilon_w = O\big(d^{-2^L}\big)$.

These error-propagation bounds translate directly into the fault-tolerant gate-count overhead when using a discrete universal set (e.g. Clifford + T). Preparing an arbitrary quantum state or block-encoding a classical matrix to precision ε requires $\mathcal{O}(\log(\frac{1}{\varepsilon}))$ T gates [2, 3]. From our multi-layer error analysis, the input (or weight) encoding error must satisfy $\varepsilon_{x/w} = O\big(d^{-2^L}\big)$ to keep the end-to-end error $\varepsilon_{\text{tot}} = \mathcal{O}(1)$ after L layers. Plugging this into the $\mathcal{O}(\log(\frac{1}{\varepsilon}))$ scaling gives a T-gate overhead of

$$
\log(\tfrac{1}{\varepsilon_{\text{tot}}}) = \log(d^{2^L}) = 2^L \log d, \quad (7)
$$

i.e., an exponential dependence on the network depth L. Hence the total T gate complexity of a L-layer CHEB-QKAN is

$$
C_{\text{tot}} = \mathcal{O}\big((2d^2)^L \log d\big). \quad (8)
$$

III. SUPPLEMENTARY NOTE 3. OUTPUT ESTIMATION OF CHEB-QKAN

In this supplementary section, we show how amplitude estimation on a suitably constructed Hadamard-test circuit yields the precision and complexity stated in Theorem 2.

THEOREM 2 (Output estimation of CHEB-QKAN). *Given access to a controlled diagonal $(1, a_x, \varepsilon_x)$-block-encoding U_x of an input vector $\boldsymbol{x} \in [-1,1]^N$, and access to $d+1$ controlled diagonal $(1, a_w, \varepsilon_w)$-block-encodings $U_{w^{(r)}}$ of weight vectors $\boldsymbol{w}^{(r)} \in [-1,1]^{NK}$, we can estimate the value $\Phi(\boldsymbol{x})_q = \frac{1}{N}\sum_{p=1}^{N}\phi_{pq}(x_p)$ of the q-th component of the* CHEB-QKAN *layer to $\left(4d\sqrt{\varepsilon_x} + \varepsilon_w + \delta\right)$-precision using $\mathcal{O}\left(d^2/\delta\right)$ applications of controlled-U_x and controlled-$U_{w^{(r)}}$ and their adjoint versions.*

Proof. By Theorem 1 in the main text, we can construct a diagonal block-encoding U_Φ such that

$$\left\| \langle 0|_{\text{aux}} U_\Phi |0\rangle_{\text{aux}} - \operatorname{diag}\left(\frac{1}{N}\sum_{p=1}^{N}\phi_{p1}(x_p), \dots, \frac{1}{N}\sum_{p=1}^{N}\phi_{pK}(x_p)\right) \right\| \le 4d\sqrt{\varepsilon_x} + \varepsilon_w \tag{9}$$

using $\mathcal{O}\left(d^2\right)$ applications of controlled-U_x and controlled-$U_{w^{(r)}}$ and their adjoint versions. Consider $\tilde{U}_\Phi := (H \otimes I_{\text{aux}+k}) C U_\Phi (H \otimes I_{\text{aux}+k})$. Applying $\tilde{U}_\Phi$ to the computational basis state $|0\rangle |0\rangle_{\text{aux}} |q\rangle_k$ yields

$$\begin{aligned}
\tilde{U}_\Phi |0\rangle |0\rangle_{\text{aux}} |q\rangle_k &= |0\rangle \left(\frac{U_\Phi + I_{\text{aux}+k}}{2}\right) |0\rangle_{\text{aux}} |q\rangle_k + |1\rangle \left(\frac{U_\Phi - I_{\text{aux}+k}}{2}\right) |0\rangle_{\text{aux}} |q\rangle_k \\
&= \left(\frac{\alpha_q + 1}{2}\right) |0\rangle |0\rangle_{\text{aux}} |q\rangle_k + \sqrt{1 - \left(\frac{\alpha_q + 1}{2}\right)^2}\, |\perp\rangle ,
\end{aligned}$$

where $|\alpha_q - \frac{1}{N}\sum_{p=1}^{N}\phi_{pq}(x_p)| \le 4d\sqrt{\varepsilon_x} + \varepsilon_w$ by Theorem 1 in the main text and $|\perp\rangle$ is orthogonal to $|0\rangle |0\rangle_{\text{aux}} |q\rangle_k$. Using amplitude estimation, we can estimate the absolute value $|\frac{\alpha_q+1}{2}|$ of the amplitude of the good state $|0\rangle |0\rangle_{\text{aux}} |q\rangle_k$ to the additive δ-precision using $\mathcal{O}\left(1/\delta\right)$ queries to $\tilde{U}_\Phi$ and $\tilde{U}_\Phi^\dagger$ [4], resulting in a total complexity of $\mathcal{O}\left(d^2/\delta\right)$ queries to controlled-U_x and controlled-$U_{w^{(r)}}$ and their adjoint versions. Since $|\alpha_q| \le 1$, it follows that $\frac{\alpha_q+1}{2} \ge 0$ and we can infer the value of α_q from the estimated amplitude. □

IV. SUPPLEMENTARY NOTE 4. MULTIVARIATE GAUSSIAN STATE PREPARATION

In this supplementary section, we show how to prepare a multivariate Gaussian state with the use of the grid encoding in Lemma 3 and prove the complexity stated in Theorem 4.

LEMMA 3 (Multivariate grid encoding). *Let $G_D = \operatorname{diag}\big(x_{(i_1,\dots,i_D)}\big)_{(i_1,\dots,i_D)\in\{0,\dots,2^n-1\}^D}$, where*

$$x_{(i_1,\dots,i_D)} = \big(-1 + i_1\, s,\ -1 + i_2\, s,\ \dots,\ -1 + i_D\, s\big) \in [-1,1]^D, \tag{10}$$

be a uniform (vectorized) D-dimensional grid on $[-1,1]^D$ with step size $s = \frac{2}{2^n-1}$ in every direction. The dimension of G_D is $D\,2^{nD}$. Then,

$$G_1 = \sum_{i=1}^{n}\Big(\frac{2^{i-1}}{2^n-1}\Big) I_{i-1} \otimes XZX \otimes I_{n-i} \quad \textit{and} \quad G_D = \sum_{j=1}^{D} I_{n(j-1)} \otimes G_1 \otimes I_{n(D-j)} \otimes |j\rangle\langle j| , \tag{11}$$

and we can create a $(1, D\lceil \log n\rceil, 0)$-block-encoding of G_D using $\mathcal{O}(Dn(\log n + \log D))$ two-qubit gates.

THEOREM 4. *We can prepare a Dn-qubit quantum state $|\psi\rangle$ with amplitudes corresponding to a D-dimensional Gaussian distribution on a regular square grid of size $(2^n)^D$ such that*

$$\left\| |\psi\rangle \; - \; \frac{1}{\widetilde{F}_{\text{exp}}} \sum_{i_1,\dots,i_D}^{2^n} \exp(-\tfrac{\beta}{2}\sum_{j=1}^{D} x_{i_j}^2)\ |i_1,\dots,i_D\rangle \right\|_2 \le \delta, \tag{12}$$

where $\widetilde{F}_{\exp}$ *normalizes the target state. The procedure succeeds with arbitrarily high probability by using* $\widetilde{\mathcal{O}}\big(\beta^{\frac{D}{4}+\frac{1}{2}}\, n\, \log\frac{1}{\delta}\big)$ *two-qubit gates and* $D\lceil\log n\rceil + \lceil\log D\rceil + 4$ *ancilla qubits.*

A. Multivariate grid encoding

In the following, we extend the univariate grid encoding in Lemma IV.1 to D-dimensions to encode a multivariate grid, thus proving Lemma 3.

Lemma IV.1 (Univariate grid encoding – [5]). *Let* $G_1 = \mathrm{diag}(-1 + j\, s)_{j=0,\ldots,2^n-1}$ *be a uniform grid on the interval* $[-1, 1]$ *with step size* $s = \frac{2}{2^n-1}$*. Then,*

$$G_1 = \sum_{i=1}^{n} \Big(\frac{2^{i-1}}{2^n-1}\Big)\, I_{i-1} \otimes XZX \otimes I_{n-i} \tag{13}$$

and we can create a $(1, \lceil\log n\rceil, 0)$*-block-encoding of* G_1 *using* $\mathcal{O}(n\log n)$ *two-qubit gates.*

Proof of Lemma 3. Let $m = \lceil\log n\rceil$ and

$$A \;=\; \sum_{i=1}^{n} \sqrt{\frac{2^{i-1}}{2^n-1}}\;\, |i\rangle\langle 0|_m \;+\; \big(\text{u.c.}\big), \tag{14}$$

be a state-preparation unitary acting on m ancilla qubits, where (u.c.) denotes an arbitrary unitary complement. For each $j = 1, \ldots, D$ define

$$B_j = \sum_{i=1}^{n} \underbrace{I_{m(j-1)} \otimes |i\rangle\langle i| \otimes I_{m(D-j)}}_{Dm} \otimes \underbrace{I_{n(j-1)} \otimes I_{i-1} \otimes XZX \otimes I_{n-i} \otimes I_{n(D-j)}}_{Dn} + \big(\text{u.c.}\big) \tag{15}$$

with underbraces denoting the numbers qubits the operators are acting on. Let $k = \lceil\log D\rceil$. Then, the unitary

$$U_{G_D} = \Big(\underbrace{A^\dagger \otimes \cdots \otimes A^\dagger}_{D\ \text{times}} \otimes I_{Dn} \otimes I_k\Big)\Big(\sum_{j=1}^{D} B_j \otimes |j\rangle\langle j| \;+\; \big(\text{u.c.}\big)\Big)\Big(\underbrace{A \otimes \cdots \otimes A}_{D\ \text{times}} \otimes I_{Dn} \otimes I_k\Big) \tag{16}$$

is a $(1, D\, m, 0)$–block-encoding of G_D because

$$\begin{aligned}
\langle 0|_{Dm}\, U_{G_D}\, |0\rangle_{Dm} &= \Big(\langle 0|_{Dm}\, A^{\dagger\otimes D} \otimes I_{Dn} \otimes I_k\Big)\, \Big(\sum_{j=1}^{D} B_j \otimes |j\rangle\langle j| \;+\; \big(\text{u.c.}\big)\Big)\, \Big(A^{\otimes D}\, |0\rangle_{Dm} \otimes I_{Dn} \otimes I_k\Big) \\
&= \sum_{j=1}^{D} I_{n(j-1)} \otimes \Big(\sum_{i=1}^{n} \langle 0|_m\, A^\dagger\, |i\rangle\langle i|\, A\, |0\rangle_m\;\, I_{i-1} \otimes XZX \otimes I_{n-i}\Big) \otimes I_{n(D-j)} \otimes |j\rangle\langle j| \\
&= \sum_{j=1}^{D} I_{n(j-1)} \otimes \Big(\sum_{i=1}^{n} \Big(\frac{2^{i-1}}{2^n-1}\Big)\, I_{i-1} \otimes XZX \otimes I_{n-i}\Big) \otimes I_{n(D-j)} \otimes |j\rangle\langle j| \\
&= \sum_{j=1}^{D} I_{n(j-1)} \otimes G_1 \otimes I_{n(D-j)} \otimes |j\rangle\langle j|\,.
\end{aligned}$$

Finally, one verifies that $G_D \;=\; \sum_{j=1}^{D} I_{n(j-1)} \otimes G_1 \otimes I_{n(D-j)} \otimes |j\rangle\langle j|$ because

$$\langle i_1, i_2, \ldots, i_D|\; G_D\; |i_1, i_2, \ldots, i_D\rangle = \sum_{j=1}^{D} \langle i_j|\; G_1\; |i_j\rangle\;\; |j\rangle\langle j|$$

$$= \big(-1 + i_1\, s,\ -1 + i_2\, s,\ \ldots,\ -1 + i_D\, s\big)$$
$$= x_{(i_1,\ldots,i_D)}.$$

The state preparation unitary A can be constructed using $\mathcal{O}(n)$ two-qubit gates using a state preparation circuit with real coefficients [6]. The operator $\sum_{j=1}^{D} B_j \otimes |j\rangle\langle j| + \big(\text{u.c.}\big)$ is composed of D k-qubit-controlled B_j operators applied in sequence, where each B_j requires n m-qubit controlled Z gates. Since an m-qubit-controlled Z gate can be implemented using $\mathcal{O}(m)$ two-qubit gates [7, 8], the total two-qubit gate cost of U_{G_D} is $\mathcal{O}(Dn(m+k)) = \mathcal{O}(Dn(\log n + \log D))$. □

B. Polynomial approximation to exponential decay $e^{-\beta(x+1)}$

One can show by simple truncation the Taylor series expansion of $e^{-\beta(x+1)}$ on the interval $[-1,1]$ that $e^{-\beta(x+1)}$ can be approximated to ε precision using a polynomial of degree $d = \mathcal{O}\big(\sqrt{\beta}\log\frac{1}{\varepsilon}\big)$. Lemma IV.2 makes this precise.

Lemma IV.2 (Adapted from [9]). $\forall \beta > 0, \varepsilon \in (0, 1/2]$, *there exists a polynomial* P_d *of degree* $d = \lceil\sqrt{2\lceil\max[\beta e^2, \log(2/\varepsilon)]\rceil\log(4/\varepsilon)}\rceil$ *such that*

$$\max_{x\in[-1,1]} |P_d(x) - e^{-\beta(x+1)}| \leq \varepsilon.$$

This polynomial can be efficiently decomposed without error in the Chebyshev basis, and is hence implementable without error via QSVT with $\mathcal{O}\big(\sqrt{\beta}\log\frac{1}{\varepsilon}\big)$ queries to the block-encoding of $\Phi(G_D)$ [10–13].

C. Bounding the filling fraction of a D-dimensional Gaussian

We demonstrate in the main text that the block-encoding of $\Phi_2(G_D)$ yields an approximation of the target D-dimensional Gaussian when applied to the uniform superposition state. The resulting state is subnormalized and needs to be amplified using amplitude amplification, which requires knowing a lower bound on the success probability p, which is given by the (discretized) ℓ_2 filling fraction $\widetilde{F}^2_{P_d}$ of the approximating polynomial $P_d(x)$ on $x \in [-1,1]$:

$$p \;=\; \|\Phi_2(G_D)\,|+\rangle_{Dn}\|_2^2 \;=\; \frac{1}{2^{Dn}}\sum_{i_1,\ldots,i_D} P_d\Big(\tfrac{2}{D}\sum_{j=1}^{D} x_{i_j}^2 - 1\Big)^2 \;=\; \widetilde{F}^2_{P_d}. \tag{17}$$

In the following, we will bound $\widetilde{F}_{P_d}$ by first approximating it with its "target" version $\widetilde{F}_{\exp}$, defined as

$$\widetilde{F}_{P_d} \;\approx\; \widetilde{F}_{\exp} \;=\; \Big(\frac{1}{2^{Dn}}\sum_{i_1,\ldots,i_D}\exp\big(-\beta\sum_{j=1}^{D} x_{i_j}^2\big)\Big)^{\frac{1}{2}} \;=\; \Big(\frac{1}{2^n}\sum_{i=0}^{2^n-1}\exp\big(-\beta x_i^2\big)\Big)^{\frac{D}{2}} \tag{18}$$

and then approximating $\widetilde{F}_{\exp}$ with its continuous version, for which the lower bound can be computed analytically:

$$\widetilde{F}_{\exp} \;\approx\; F_{\exp} \;=\Big(\frac{1}{2^D}\int_{[-1,1]^D} dx_1\cdots dx_D\,\exp\big(-\sum_{i=1}^{D}\beta_i\,x_i^2\big)\Big)^{\frac{1}{2}} \;=\; \Big(\frac{1}{2}\int_{[-1,1]} dx\,\exp\big(-\beta x^2\big)\Big)^{\frac{D}{2}}. \tag{19}$$

First, we consider the approximation error from the polynomial expansion:

$$\Big|\widetilde{F}^2_{\mathrm{P_d}} - \widetilde{F}^2_{\exp}\Big| = \left|\Big(\frac{1}{2^n}\Big)^D\sum_{i_1,\ldots,i_D} P_d\Big(\tfrac{2}{D}\sum_{j=1}^{D} x_{i_j}^2 - 1\Big)^2 - \Big(\frac{1}{2^n}\Big)^D\sum_{i_1,\ldots,i_D}\exp\big(-\beta\sum_{j=1}^{D} x_{i_j}^2\big)\right|$$

$$\le \Big(\frac{1}{2^n}\Big)^D \sum_{i_1,\ldots,i_D} \left| P_d\Big(\tfrac{2}{D}\sum_{j=1}^{D} x_{i_j}^2 - 1\Big)^2 - \exp\big(-\beta \sum_{j=1}^{D} x_{i_j}^2\big) \right|.$$

By the polynomial approximation (Lemma IV.2), we are guaranteed that $\max_{x\in[-1,1]} |P_d(x) - e^{-\frac{D\beta}{4}(x+1)}| \le \varepsilon$. Consequently, $|P_d(x)^2 - e^{-\frac{D\beta}{2}(x+1)}| \le 2\varepsilon \cdot \max_{x\in[-1,1]}\{P_d(x), e^{-\frac{D\beta}{4}(x+1)}\} \le 2\varepsilon$. As a result, we obtain

$$\left| \widetilde{F}_{\mathrm{P_d}}^2 - \widetilde{F}_{\mathrm{exp}}^2 \right| \le 2\varepsilon. \tag{20}$$

Second, we consider the discretization error for the filling fraction, i.e., the difference between the discretized and continuous versions $\widetilde{F}_{\mathrm{exp}}$ and F_{exp}. Recall the standard result on Riemann sums:

Lemma IV.3 (Error bound on the Riemann sum). *Suppose that $f\colon [a,b] \to \mathbb{R}$ is twice continuously differentiable. Let $x_i = ((b-a)i/N + a)$, then*

$$\left| \frac{b-a}{N} \sum_{i=0}^{N-1} f(x_i) - \int_a^b f(x)dx \right| \le \frac{(b-a)^2}{2N} |f'(x)|_{\max}^{x\in[a,b]}.$$

Here we consider the left Riemann sum for bounds, hence, when creating the multivariate grid, we need to replace the univariate grids to fit the grid points of the left Riemann sum. This can be obtained by rescaling the univariate grid by $\frac{2^n-1}{2^n}$ and shifting downwards by $\frac{1}{2^n}$, which can be a weighted sum of the univariate grid and identity via LCU [12, 14].

For the one-dimensional case, the discretization error for the filling fraction can be found as follows:

$$\left| \frac{1}{2^n} \sum_{i=0}^{2^n-1} e^{-\beta x_i^2} - \frac{1}{2}\int_{-1}^{1} e^{-\beta x^2} dx \right| \le \frac{1}{2^n}\sqrt{\frac{2\beta}{e}}, \tag{21}$$

since the first derivative is bounded by $|(e^{-\beta x^2})'|_{\max} = \sqrt{\frac{2\beta}{e}}$. To extend the bound to the D-dimensional case, let $a = \frac{1}{2^n}\sum_{i=0}^{2^n-1} e^{-\beta x_i^2}$ and $b = \frac{1}{2}\int_{-1}^{1} e^{-\beta x^2} dx$. Then,

$$|a^D - b^D| = |a-b| \sum_{k=1}^{D} a^{D-k} b^{k-1} \le |a-b| \cdot D \quad \text{because} \quad a, b \le 1. \tag{22}$$

Consequently, $\left| \widetilde{F}_{\mathrm{exp}}^2 - F_{\mathrm{exp}}^2 \right| = |a^D - b^D| \le \frac{D}{2^n}\sqrt{\frac{2\beta}{e}}$. Putting it all together, we have

$$\left| \widetilde{F}_{P_d}^2 - F_{\mathrm{exp}}^2 \right| \le \left| \widetilde{F}_{P_d}^2 - \widetilde{F}_{\mathrm{exp}}^2 \right| + \left| \widetilde{F}_{\mathrm{exp}}^2 - F_{\mathrm{exp}}^2 \right| \le 2\varepsilon + \tfrac{D}{2^n}\sqrt{\tfrac{2\beta}{e}}. \tag{23}$$

As a result, we have a lower bound on $\widetilde{F}_{P_d}$ in terms of F_{exp}:

$$\widetilde{F}_{P_d} \ge F_{\mathrm{exp}}\sqrt{1 - \tfrac{1}{F_{\mathrm{exp}}^2}(2\varepsilon + \tfrac{D}{2^n})} \ge F_{\mathrm{exp}} - \tfrac{1}{F_{\mathrm{exp}}}(2\varepsilon + \tfrac{D}{2^n}), \tag{24}$$

assuming $2\varepsilon + \frac{D}{2^n} \le F_{\mathrm{exp}}^2$. Having a bound in terms of F_{exp} is useful because F_{exp} can be lower-bounded analytically:

Lemma IV.4 (Continuous filling fraction of a D-dimensional Gaussian). *Let*

$$F_{\mathrm{exp}}^2 := \frac{1}{2^D}\int_{[-1,1]^D} dx_1 \cdots dx_D \, e^{-\sum_{i=1}^{D} \beta_i x_i^2} \tag{25}$$

be the ℓ_2 filling fraction of a D-dimensional diagonal Gaussian on the interval $[-1,1]^D$. Then, for $\beta \ge 1$,

$$F_{\mathrm{exp}}^2 \ge \Big(\frac{2}{3}\Big)^D \prod_{i=1}^{D} \beta_i^{-\frac{1}{2}}. \tag{26}$$

Proof. The proof of the D-dimensional case is an extension of the one-dimensional result in Appendix E, Lemma 4 of [15]. For $x \in [-1,1]$ it holds that $\exp(-\beta x^2) \geq 1 - \beta x^2$. For $\beta \geq 1$, we bound $\frac{1}{2}\int_{-1}^{1} e^{-\beta x^2}\,dx \geq \int_{-1/\sqrt{\beta}}^{1/\sqrt{\beta}} e^{-\beta x^2}\,dx \geq \int_{-1/\sqrt{\beta}}^{1/\sqrt{\beta}} (1-\beta x^2)\,dx = \frac{2}{3\sqrt{\beta}}$. Therefore for the D-dimensional product,

$$F_{\exp}^2 = \prod_{i=1}^{D} \frac{1}{2}\int_{-1}^{1} e^{-\beta_i x_i^2}\,dx_i \;\geq\; \prod_{i=1}^{D} \frac{2}{3\sqrt{\beta_i}} = \left(\frac{2}{3}\right)^D \prod_{i=1}^{D} \beta_i^{-\frac{1}{2}}. \tag{27}$$

In particular, if $\beta_i = \beta$ for all i, then $F_{\exp} \;\geq\; \left(\frac{2}{3\sqrt{\beta}}\right)^{\frac{D}{2}}$ for $\beta \geq 1$. For $\beta < 1$, one can similarly show that $F_{\exp} \;\geq\; (2/3)^{\frac{D}{2}}$, independent of β. □

Using Lemma IV.4, we can bound

$$\frac{1}{\widetilde{F}_{P_d}} \leq \frac{2}{F_{\exp}} = \mathcal{O}\big((\tfrac{3}{2})^{\frac{D}{2}} \beta^{\frac{D}{4}}\big). \tag{28}$$

D. Gate and qubit complexity of Gaussian state preparation

In this section, we calculate the two-qubit gate complexity and the number of ancilla qubits throughout our construction, thereby proving Theorem 4.

1. *Step 1 – Grid encoding:* Implementing U_{G_D} requires $\mathcal{O}\big(Dn(\log n + \log D)\big)$ two-qubit gates, where U_{G_D} is acting on $D(n + \lceil \log n \rceil) + \lceil \log D \rceil$ qubits, according to Lemma 3.

2. *Step 2 – First layer:* The block-encoding of $\Phi_1(G_D)$ requires $\mathcal{O}(1)$ applications of U_{G_D} and $\mathcal{O}(D \log n)$ additional two-qubit gates. It also consumes one additional ancilla qubit for the qubitization procedure [16].

3. *Step 3 – Second layer:* The block-encoding of $\Phi_2(G_D)$ requires $\mathcal{O}(d)$ applications of the block-encoding of $\Phi_1(G_D)$ and $\mathcal{O}\big(d \times Dn(\log n + \log D)\big)$ additional two-qubit gates, and consumes two additional ancilla qubits according to Theorem 31 of Ref. [12]. Putting it all together, the two-qubit gate cost of constructing the block-encoding of $\Phi_2(G_D)$ is $\mathcal{O}\big(d \times (D \log n + \log D)\big)$. The number of main qubits $\Phi_2(G_D)$ is acting on is Dn and the number of ancillas is $D\lceil \log n \rceil + \lceil \log D \rceil + 3$.

4. *Step 4 – Amplitude amplification:* The QSVT-based fixed-point amplitude amplification requires $\mathcal{O}\big(\frac{1}{\widetilde{F}_{P_d}}\big)$ applications of the block-encoding of $\Phi_2(G_D)$ to amplify the subnormalized state $\Phi_2(G_D)\,|+\rangle_{Dn}$ to be arbitrarily close to its normalized version $\frac{1}{\widetilde{F}_{P_d}}\Phi_2(G_D)\,|+\rangle_{Dn}$. In addition, it consumes one additional ancilla and additional $\mathcal{O}\big(\frac{1}{\widetilde{F}_{P_d}} \times Dn(\log n + \log D)\big)$ two-qubit gates for the reflection operators [12, 16]. As a result, the total two-qubit gate complexity becomes $\mathcal{O}\big(\frac{d}{\widetilde{F}_{P_d}} \times Dn(\log n + \log D)\big)$. The number of main qubits is Dn and the number of ancillas is $D\lceil \log n \rceil + \lceil \log D \rceil + 4$.

To guarantee the target accuracy δ of the state preparation, we first consider the error in the subnormalized state $\widetilde{F}_{P_d}\,|\psi\rangle = \Phi_2(G_D)\,|+\rangle_{Dn}$. By Lemma IV.2,

$$\left\|\Phi_2(G_D)\,|+\rangle_{Dn} - \sum_{i_1,\ldots,i_D}^{2^n} \exp(-\tfrac{\beta}{2}\sum_{j=1}^{D} x_{i_j}^2)\;|i_1,\ldots,i_D\rangle\right\|_2 \leq \left\|\Phi_2(G_D) - \mathrm{diag}\big(\exp(-\tfrac{\beta}{2}\sum_{j=1}^{D} x_{i_j}^2)\big)\right\|_2 \leq \varepsilon. \tag{29}$$

However, amplitude amplification amplifies not only the success probability but also the error in the solution. Let $|\psi_{\text{target}}\rangle$ denote the target quantum state

$$|\psi_{\text{target}}\rangle = \frac{1}{\widetilde{F}_{\exp}} \sum_{i_1,\ldots,i_D}^{2^n} \exp(-\tfrac{\beta}{2}\sum_{j=1}^{D} x_{i_j}^2)\;|i_1,\ldots,i_D\rangle\,. \tag{30}$$

Then, to achieve the target approximation accuracy $\||\psi\rangle - |\psi_{\text{target}}\rangle\|_2 \leq \delta$, note that

$$\||\psi\rangle - |\psi_{\text{target}}\rangle\|_2 \leq \left\||\psi\rangle - \frac{\widetilde{F}_{\exp}}{\widetilde{F}_{P_d}}|\psi_{\text{target}}\rangle\right\|_2 + \left\|\frac{\widetilde{F}_{\exp}}{\widetilde{F}_{P_d}}|\psi_{\text{target}}\rangle - |\psi_{\text{target}}\rangle\right\|_2 \leq \frac{\varepsilon}{\widetilde{F}_{P_d}} + \left|\frac{\widetilde{F}_{\exp}}{\widetilde{F}_{P_d}} - 1\right|. \tag{31}$$

Hence, the polynomial approximation accuracy ε must be chosen such that $\varepsilon + |\widetilde{F}_{\exp} - \widetilde{F}_{P_d}| \leq \widetilde{F}_{P_d} \cdot \delta$. Using the bounds obtained in Section IV C, it suffices to choose ε to satisfy

$$\varepsilon + \frac{4\varepsilon}{F_{\exp}} \leq \delta \cdot \left(F_{\exp} + \frac{1}{F_{\exp}}(2\varepsilon + \frac{D}{2^n})\right). \tag{32}$$

Rearranging for ε and assuming $\delta \leq \frac{F_{\exp}}{4}$, we obtain $\varepsilon \leq 2\delta\left(F_{\exp} + \frac{D}{2^n F_{\exp}}\right)$. Furthermore, assuming $\frac{D}{2^n} \leq F_{\exp}^2$, which holds for sufficiently large grid sizes, where $n \in \Omega(D \log \beta)$ it suffices to choose $\varepsilon \leq 4\delta F_{\exp}$. Finally, plugging in the lower bound on $F_{\exp}$ (Lemma IV.4), we can definitively set $\varepsilon = 4\delta \cdot \left(\frac{2}{3\sqrt{\beta}}\right)^{\frac{D}{2}}$. This implies that the polynomial degree $\mathcal{O}\left(\sqrt{D\beta} \log \frac{1}{\varepsilon}\right)$ becomes a function of D, β, and δ:

$$d = \mathcal{O}\left(\sqrt{D\beta}\, \log \beta^{\frac{D}{4}} \log \tfrac{1}{\delta}\right). \tag{33}$$

Finally, substituting the bound $\frac{1}{\widetilde{F}_{P_d}} = \mathcal{O}\left((\frac{3}{2})^{\frac{D}{2}} \beta^{\frac{D}{4}}\right)$ from Section IV C together with the required polynomial degree $d = \mathcal{O}\left(\sqrt{D\beta}\, \log \beta^{\frac{D}{4}} \log \frac{1}{\delta}\right)$, we obtain the announced two-qubit-gate complexity of the D-dimensional Gaussian quantum state preparation as a function of β, D, n and δ:

$$\mathcal{O}\left(\beta^{\frac{D}{4}+\frac{1}{2}} \log \beta \cdot (\tfrac{3}{2})^{\frac{D}{2}} D^{\frac{5}{2}} \log D \cdot n \log n \cdot \log \tfrac{1}{\delta}\right). \tag{34}$$

Suppressing the logarithmic factors $\log n$ and $\log \beta$, and treating D as a constant, we have the simplified form

$$\widetilde{\mathcal{O}}\left(\beta^{\frac{D}{4}+\frac{1}{2}}\, n \log \tfrac{1}{\delta}\right). \tag{35}$$

in agreement with Theorem 4.

V. SUPPLEMENTARY NOTE 5. GENERALIZED STATE PREPARATION VIA CHEB-QKAN

In the following, we prove Theorem 12 by applying the CHEB-QKAN block-encoding to the uniform superposition over the output register, and then using fixed-point amplitude amplification to boost the overlap with the target state to arbitrarily high probability.

THEOREM 12 (Multivariate state preparation via CHEB-QKAN). *Let $\varepsilon \in (0, \frac{1}{2})$. We are given access to a controlled diagonal $(1, a_x, \varepsilon_x)$-block-encoding U_x of an input vector $\boldsymbol{x} \in [-1,1]^N$, and access to $d+1$ controlled diagonal $(1, a_w, \varepsilon_w)$-block-encodings $U_{w^{(r)}}$ of weight vectors $\boldsymbol{w}^{(r)} \in [-1,1]^{NK}$. Let $\mathcal{N}^2 := \sum_{q=1}^{K}\left(\frac{1}{N}\sum_{p=1}^{N}\phi_{pq}(x_p)\right)^2$ and d be the maximal degree of Chebyshev polynomials used in parameterization of activation functions ϕ_{pq}. If $\varepsilon_x \leq \frac{\mathcal{N}^2}{144Kd^2}\varepsilon^2$ and $\varepsilon_w \leq \frac{\mathcal{N}}{3\sqrt{K}}\varepsilon$, then we can prepare a ℓ_2 normalized quantum state $|\psi\rangle$ with amplitudes corresponding to a* CHEB-QKAN *layer such that*

$$\left\||\psi\rangle - \frac{1}{\mathcal{N}}\sum_{q=1}^{K}\left(\frac{1}{N}\sum_{p=1}^{N}\phi_{pq}(x_p)\right)|q\rangle_k\right\|_2 \leq \varepsilon, \tag{36}$$

The procedure succeeds with arbitrarily high probability by using $\mathcal{O}(\sqrt{K}d^2/\mathcal{N})$ applications of controlled-U_x and controlled-$U_{w^{(r)}}$ and their adjoint versions.

Proof. By Theorem 1 in the main text, we can construct a diagonal block-encoding U_Φ such that

$$\left\| \langle 0|_{\text{aux}} U_\Phi |0\rangle_{\text{aux}} - \text{diag}\left(\frac{1}{N}\sum_{p=1}^{N}\phi_{p1}(x_p),\ldots,\frac{1}{N}\sum_{p=1}^{N}\phi_{pK}(x_p)\right)\right\| \le 4d\sqrt{\varepsilon_x} + \varepsilon_w \tag{37}$$

using $\mathcal{O}\left(d^2\right)$ applications of controlled-U_x and controlled-$U_{w^{(r)}}$ and their adjoint versions. To prepare $|\psi\rangle$, we can apply U_Φ to the state $|0\rangle_{\text{aux}} |+\rangle_k$ and measure the auxiliary qubits in the state $|0\rangle_{\text{aux}}$. Then,

$$\begin{aligned}
&\left\| \frac{1}{\sqrt{p}} \langle 0|_{\text{aux}} U_\Phi |0\rangle_{\text{aux}} |+\rangle_k - \frac{1}{\sqrt{p}}\frac{1}{\sqrt{K}}\sum_{q=1}^{K}\left(\frac{1}{N}\sum_{p=1}^{N}\phi_{pq}(x_p)\right)|q\rangle_k \right\|_2 \\
\le& \frac{1}{\sqrt{p}}\left\| \langle 0|_{\text{aux}} U_\Phi |0\rangle_{\text{aux}} - \text{diag}\left(\frac{1}{N}\sum_{p=1}^{N}\phi_{p1}(x_p),\ldots,\frac{1}{N}\sum_{p=1}^{N}\phi_{pK}(x_p)\right)\right\| \\
\le& \frac{1}{\sqrt{p}}\left(4d\sqrt{\varepsilon_x} + \varepsilon_w\right),
\end{aligned}$$

where $\sqrt{p} = \| \langle 0|_{\text{aux}} U_\Phi |0\rangle_{\text{aux}} |+\rangle_k \|_2$ is the square root of the probability of successful outcome such that $|\sqrt{p} - \frac{\mathcal{N}}{\sqrt{K}}| \le 4d\sqrt{\varepsilon_x} + \varepsilon_w$. We can boost this probability to an arbitrarily high success probability by using $\mathcal{O}(1/\sqrt{p})$ amplitude amplification steps [17].

To bound the total error within $\varepsilon \in (0, \frac{1}{2})$, let $\gamma \ge 4d\sqrt{\varepsilon_x} + \varepsilon_w$. Then we upper bound the total error by ε so that $\frac{4d\sqrt{\varepsilon_x}+\varepsilon_w}{\sqrt{p}} \le \frac{\gamma}{\mathcal{N}/\sqrt{K}-\gamma} \le \varepsilon$. Rearranging the terms, we find that $\gamma \le \frac{\mathcal{N}}{\sqrt{K}}\frac{\varepsilon}{1+\varepsilon}$. Note that given $\varepsilon \le \frac{1}{2}$, $\frac{2}{3} \le \frac{1}{1+\varepsilon}$. Set $\gamma = \frac{2}{3}\frac{\mathcal{N}}{\sqrt{K}}\varepsilon$. As such, if $\varepsilon_x \le \frac{\mathcal{N}^2}{144Kd^2}\varepsilon^2$ and $\varepsilon_w \le \frac{\mathcal{N}}{3\sqrt{K}}\varepsilon$, then $4d\sqrt{\varepsilon_x} \le \frac{\mathcal{N}}{3\sqrt{K}}\varepsilon$ and the ℓ_2 error of the prepared state is upper bounded by $\frac{4d\sqrt{\varepsilon_x}+\varepsilon_w}{\sqrt{p}} \le \varepsilon$.

Lastly, to calculate the runtime $\mathcal{O}(1/\sqrt{p})$, we note that $\sqrt{p}$ is lower bounded by $\frac{\mathcal{N}}{\sqrt{K}} - \gamma$ which can then be lower bounded by $\frac{\mathcal{N}}{\sqrt{K}} - \frac{2}{3}\frac{\mathcal{N}}{\sqrt{K}}\varepsilon$ and furthermore, $\frac{2}{3}\frac{\mathcal{N}}{\sqrt{K}}$. Hence, the total complexity can be found to be $\mathcal{O}(\sqrt{K}d^2/\mathcal{N})$. □

REFERENCES

[1] M. A. Nielsen and I. L. Chuang, *Quantum Computation and Quantum Information* (Cambridge University Press, 2010).

[2] N. J. Ross and P. Selinger, Optimal ancilla-free Clifford+T approximation of z-rotations, Quantum Info. Comput. **16**, 901–953 (2016).

[3] B. D. Clader, A. M. Dalzell, N. Stamatopoulos, G. Salton, M. Berta, and W. J. Zeng, Quantum resources required to block-encode a matrix of classical data, IEEE Trans. Quantum Eng. **3**, 1 (2022).

[4] G. Brassard, P. Høyer, M. Mosca, and A. Tapp, Quantum amplitude amplification and estimation, in *Quantum computation and information*, Contemporary Mathematics, Vol. 305 (American Mathematical Society, Providence, RI, USA, 2002) pp. 53–74.

[5] M. Rosenkranz, E. Brunner, G. Marin-Sanchez, N. Fitzpatrick, S. Dilkes, Y. Tang, Y. Kikuchi, and M. Benedetti, Quantum state preparation for multivariate functions, Quantum **9**, 1703 (2025).

[6] V. Bergholm, J. J. Vartiainen, M. Möttönen, and M. M. Salomaa, Quantum circuits with uniformly controlled one-qubit gates, Phys. Rev. A **71**, 052330 (2005).

[7] J. Nie, W. Zi, and X. Sun, Quantum circuit for multi-qubit Toffoli gate with optimal resource (2024), arXiv:2402.05053 [quant-ph].

[8] A. J. da Silva and D. K. Park, Linear-depth quantum circuits for multiqubit controlled gates, Phys. Rev. A **106**, 042602 (2022).

[9] S. Sachdeva, N. K. Vishnoi, *et al.*, Faster algorithms via approximation theory, Found. Trends Theor. Comput. Sci. **9**, 125 (2014).

[10] A. M. Childs, R. Kothari, and R. D. Somma, Quantum algorithm for systems of linear equations with exponentially improved dependence on precision, SIAM J. Comput. **46**, 1920–1950 (2017).

[11] G. H. Low and I. L. Chuang, Optimal Hamiltonian simulation by quantum signal processing, Phys. Rev. Lett. **118**, 010501 (2017).
[12] A. Gilyén, Y. Su, G. H. Low, and N. Wiebe, Quantum singular value transformation and beyond: exponential improvements for quantum matrix arithmetics, in *Proceedings of the 51st Annual ACM SIGACT Symposium on Theory of Computing*, STOC 2019 (Association for Computing Machinery, New York, NY, USA, 2019) pp. 193–204.
[13] E. Tang and K. Tian, A CS guide to the quantum singular value transformation, in *2024 Symposium on Simplicity in Algorithms (SOSA)* (Society for Industrial and Applied Mathematics, 2024) p. 121–143.
[14] A. M. Childs and N. Wiebe, Hamiltonian simulation using linear combinations of unitary operations, Quantum Information and Computation **12**, 10.26421/qic12.11-12 (2012).
[15] S. McArdle, A. Gilyén, and M. Berta, Quantum state preparation without coherent arithmetic (2022), arXiv:2210.14892 [quant-ph].
[16] L. Lin, Lecture notes on quantum algorithms for scientific computation (2022), arXiv:2201.08309 [quant-ph].
[17] T. J. Yoder, G. H. Low, and I. L. Chuang, Fixed-point quantum search with an optimal number of queries, Phys. Rev. Lett. **113**, 210501 (2014).
